# Mental Health Problems among Children One-Year after Sichuan Earthquake in China: A Follow-up Study

**DOI:** 10.1371/journal.pone.0014706

**Published:** 2011-02-23

**Authors:** Mingxin Liu, Li Wang, Zhanbiao Shi, Zhen Zhang, Kan Zhang, Jianhua Shen

**Affiliations:** 1 Key Laboratory of Mental Health, Institute of Psychology, Chinese Academy of Sciences, Beijing, China; 2 State Key Laboratory of Brain and Cognitive Science, Institute of Psychology, Chinese Academy of Sciences, Beijing, China; 3 Department of Psychiatry, University of Toronto, Toronto, Canada; RAND Corporation, United States of America

## Abstract

**Background:**

On May 12, 2008, a destructive earthquake registering 8.0 on the Richter scale struck Sichuan Province, southwest China. Beichuan County was the epicenter which was one of the areas nearly completely destroyed by the earthquake. In Beichuan, about 15000 people died and 3000 people were missing. Specially, the earthquake took 1587 students' and 214 teachers' lives from the elementary and middle schools there. The main purpose of the study was to provide a better understanding of mental health problems and associated risk factors among children after earthquake.

**Method:**

Three hundred and thirty grades 3–5 children completed the questionnaire of disaster –related experience and the Trauma Symptom Checklist for Children-Alternate Version (TSCC-A). The first survey was carried out six months after the earthquake, and the second one was carried out six months later. The measurements and methodology applied in the two sessions were identical.

**Results:**

The prevalence rates of the problems at two time-points were 23.3% and 22.7% for anxiety, 14.5% and 16.1% for depression, and 11.2% and 13.4% for PTSD, respectively. Among demographic variables, no significant age difference existed, while it was found that 6 months after the earthquake, symptoms of anxiety, depression and PTSD were significantly more common among students in grades 4 and 5 than those in grade 3, Initial exposure to death, bereavement and extreme fear were significant predictive factors for the occurrence of anxiety, depression and PTSD.

**Conclusions:**

Findings of this study suggest that posttraumatic mental health problems after natural disaster in children may have reached epidemic proportions and remain high for a long period. Psychologist and social workers should pay more attention to children who experienced more traumatic stresses and provide appropriate mental health interventions. Implications and limitations of these findings were discussed.

## Introduction

On May 12, 2008, a destructive earthquake registering 8.0 on the Richter scale struck Sichuan Province, southwest China. The earthquake caused extensive damage and heavy losses; it took 69227 lives, injured 374,643, led 7,923 missing, and made about 4.8 million homeless (official figures as of September 25, http://www.512gov.cn/GB/123057/8074265.html). Beichuan County (one of the counties in Sichuan; it is the location of data collection for this study) was the epicenter. The county was one of the areas nearly completely destroyed by the earthquake. Almost all the buildings, including houses, working places, schools, hospitals were ruined. In Beichuan, about 15000 people died and 3000 people were missing. Specially, the earthquake took 1587 students' and 214 teachers' lives from the elementary and middle schools there.

It has been reported that natural disasters bring physical and psychological stresses to the survivors[1]. Findings of most disaster-related studies suggest that a high prevalence rate of psychiatric problems among disaster-exposed youth could possibly lead to long-term mental health consequences, because they are generally more vulnerable than adults to trauma [2], [3]. Studies also found that younger children were more susceptible to the negative consequences of multiple risks than older ones [4], [5].

For children, as for adults, the psychopathology following disasters, such as earthquakes, hurricanes, and war, takes the forms of anxiety disorders, posttraumatic stress disorder (PTSD), panic, phobias and depression [6], [7]. Studies suggested that high prevalence of mental health problems existed among children who were directly exposed to trauma events. For example, 2 months after tsunami in southern Thailand, 10.5% children had PTSD and 8.4% developed depression; 3 months after the 1999 earthquake in Ano Liosia, Greece, 4.5% had PTSD and 13.9% had depression; and 6 months after the 9/11 terrorist attacks in New York City, 18.4% children developed depression [5], [8], [9]. Posttraumatic mental health problems in children may reach epidemic proportions, remain high for a prolonged period, and jeopardize the well-being of children populations of a large region [9], [10], [11].

Although some information on the effects of traumatic events on mental health in human beings worldwide is available, few follow-up studies regarding mental health problems in children in Asian following disasters have been published. Systematic investigating traumatic symptoms in children after such a disaster may provide critical information for rational managements of public mental health. Current study aimed to provide a better understanding of mental health problems and associated risk factors among children after earthquake.

## Methods

### Participants

Sampling was conducted in the largest camp elementary school in Beichuan County. Students in the camp school were from Qushan Town (a town in Beichuan County) School that was completely destroyed. Three hundred eighty-nine (37%) students in the original Qushan Town School died in the earthquake.

Three hundred and thirty students ranged from grade 3 to grade 5 participated in both of the investigations, which were performed six months and one year after the earthquake. Gender of the sample was approximately evenly distributed (*χ^2^* = 0.000, *p* = 1.000); the mean (*sd*) age was 10.36 (0.98) years. The sample consisted of 97 (29.4%) students in grade 3, 83 (25.2%) in grade 4 and 150 (45.5%) in grade 5.

### Measures

Demographic data included age, gender and grade. Exposure to the disaster was assessed by asking the participants the following questions: (a) whether they were trapped during the earthquake; (b) whether they were injured during the earthquake; (c) whether their family members were killed during the earthquake; (d) whether they witnessed a death of someone during or immediately after the earthquake, and (e) whether they felt intense fearful during the earthquake.

Trauma Symptom Checklist for Children-Alternate Version (TSCC-A) [12] was used to evaluate traumatic symptoms in the participants. The TSCC-A is the most wildly used test for children in trauma-related studies and practices [13]. And this instrument was verified in different races and showed cross-culture adaptation [2], [14]. It is a shortened version of TSCC that excludes the 10 items that comprise the Sexual Concerns scale and one critical item relating to sexual issues. TSCC is adopted when sexual victimization is found. This 44-item TSCC-A scale is appropriate for children who are 8–16 years of age, and it is recommended to be used in school settings. The TSCC-A consists of five subscales: anxiety, depression, anger, PTSD and dissociation. In current study, the subscales of anxiety, depression and PTSD were selected to assess posttraumatic mental health problems. The participants were asked to rate the frequency of each item in accordance with a 4-point scale ranging from 0 (never) to 3 (almost all the time). Following the criteria provided in the conversion tables of the manual, we transformed the raw scale scores into standardized T scores. Higher T scores reflect greater severity of symptomatology. A T score of 65 or higher for any of the 5 clinical subscales is considered having clinical significance. Cronbach's alpha coefficients were computed using the current sample: Anxiety (α = 0.81), Depression (α = 0.83), PTSD (α = 0.85).

### Ethics Statement

This study was approved by the ethics committees of the Institute of Psychology, Chinese Academy of Sciences. Informed written consent was obtained from children's guardians, together with oral approvals from participants, before the testing session according to the Declaration of Helsinki. A trained research assistant administered the tests in the school.

### Procedure

The investigators included clinical psychologists and psychiatrists. As mentioned, the first survey was carried out six months after the earthquake, and the second one was carried out six months later. The measurements and methodology applied in the two sessions were identical.

### Data analysis

Univariate descriptive statistics were computed for sample characteristics (sex, grade, age), trauma exposure indicators (being tapped, being injured, bereavement, witnessing death, initial fear), and prevalence rates for symptoms of anxiety, depression and PTSD, Chi-squared tests were used to evaluate differences in categorical variables and *t* tests were used to evaluate differences in continuous variables. Bivariate associations between traumatic symptoms (anxiety, depression and PTSD) and each of the other variables were evaluated with regression analyses. Those variables with *p* values of <0.05 in bivariate analysis were included in a simultaneous multivariate regression model to evaluate the significance of each predictor after controlling all the other predictors. All analyses were conducted with SPSS version 15.0.

## Results

As recommended by the manual of the TSCC-A, a T score of 65 or higher on a subscale was considered having clinical significance. This was used to identify whether a participant has anxiety, depression, or probable PTSD. According to this criterion, participants were classified as probable positive cases or probable negative cases. Table 1 shows the changes of traumatic symptoms from 6 months to 12 months after the earthquake. All the changes of the prevalence rates of probable anxiety (*p* = 0.915), probable depression (*p* = 0.783) and probable PTSD (*p* = 0.721) between the two time points were not statistically significant. Although the number of children who became clinically symptomatic was higher than those who shifted out of this status during this period, these differences are not significant.

**Table 1:** The changes of traumatic symptoms from 6-month to 12-month after the earthquake (N = 330).

	6-month	12-month	Persistent health^a^	Delayed P^b^	Persistent P^c^	Recovery^d^
Anxiety	77(23.3%)	75(22.7%)	210 (63.6%)	43 (13%)	32 (9.7%)	45 (13.6%)
Depression	48(14.5%)	53(16.1%)	253 (76.7%)	29 (8.8%)	24 (7.3%)	24 (7.3%)
PTSD	37(11.2%)	44(13.4%)	262 (79.4%)	31 (9.4%)	13 (3.9%)	24 (7.3%)

a. Persistent health: Negative in both time points.

b. Delayed P: Turn negative to positive.

c. Persistent P: Positive in both time point.

d. Recovery: Turn positive to negative.

Abbreviation: PTSD = Posttraumatic stress disorder.

To further examine the changes of the severity of traumatic symptoms, paired sample t test was conducted. Tests of significance were also accompanied by effect-size statistics, which approximate the size and thus the practical importance of the difference. The results are summarized in Table 2. paired sample t test showed the statistical significance in severity of symptoms of depression and probable PTSD, however, the small effect size showed that the severity of symptoms of anxiety, depression and PTSD at the time point of 12 months was similar with those in 6 months after the earthquake,

**Table 2:** Paired samples test for the severity of traumatic symptoms.

	Half a year	One year	t	df	p	Effect size
Anxiety	54.57±12.54	55.20±13.02	−0.85	329	0.391	0.05
Depression	50.36±12.54	52.51±12.75	−2.94	329	0.003**	0.16
PTSD	51.19±10.57	52.70±10.38	−2.51	329	0.013*	0.15

*p<0.05,

**p<0.01,

***p<0.001.

Information on trauma exposure was based on the self-reported experiences about the earthquake: 108 (32.7%) subjects were trapped during the earthquake; 138 (41.8%) reported having physical injuries; 258 (78.2%) experienced family members' death due to the earthquake; 138 (42.2%) witnessed dead bodies; and 194 (58.8%) had extreme fear in the earthquake.

In order to examine the effects of demographic and trauma exposure variables on symptoms of anxiety, depression, and PTSD at 6 months and 12 months after the earthquake, the bivariate analysis was conducted. The results have been showed in Tables 3, 4 and 5.

**Table 3:** Bivariate analysis of effects of demographic and trauma exposure variables on symptoms of anxiety at 6-month and 12-month after the Sichuan earthquake.

	Anxiety 1				Anxiety 2		
	Variable	No.(%)	OR(95%CI)	p	No.(%)	OR(95%CI)	p
Sex	Boy	36(21.8)	1	0.515	38(23)	1	0.895
	Girl	41(24.8)	1.19(0.71–1.98)		37(22.4)	0.97(0.58–1.62)	
Grade	3rd	13(13.4)	1	0.009**	20(20.6)	1	0.735
	4th	19(22.9)	1.92(0.88–4.17)		18(21.7)	1.07(0.52–2.18)	
	5th	45(30)	2.77(1.40–5.47)		37(24.7)	1.26(0.68–2.34)	
Age	8–9 y	16(15.4)	1	0.059	23(22.1)	1	0.869
	10–11 y	60(27.5)	1.90(0.97–3.69)		50(22.9)	1.05(0.60–1.84)	
Being trapped	No	46(20.7)	1	0.109	51(23)	1	0.879
	Yes	31(28.7)	1.54(0.91–2.61)		24(22.2)	0.96(0.55–1.66)	
Being injured	No	33(17.1)	1	0.002**	39(20.2)	1	0.196
	Yes	44(32.1)	2.29(1.36–3.85)		36(26.3)	1.41(0.83–2.36)	
Bereavement	No	11(15.3)	1	0.071	13(18.1)	1	0.286
	Yes	66(25.6)	1.91(0.95–3.84)		62(24)	1.44(0.74–2.79)	
Witness death	No	35(18.3)	1	0.009**	35(18.3)	1	0.024*
	Yes	42(30.4)	1.95(1.16–3.27)		40(29)	1.82(1.08–3.06)	
Extreme Fear	No	28(20.6)	1	0.324	13(9.6)	1	0.000***
	Yes	49(25.3)	1.33(0.77–2.21)		62(32)	4.44(2.33–8.48)	

*p<0.05,

**p<0.01,

***p<0.001.

Abbreviation: No. = number

Anxiety 1 and Anxiety 2 represent Anxiety at 6-month and Anxiety at 12-month, respectively.

**Table 4:** Bivariate analysis of effects of demographic and trauma exposure variables on symptoms of depression at 6-month and 12-month after the Sichuan earthquake.

	Depression 1				Depression 2		
	Variable	No.(%)	OR(95%CI)	p	No.(%)	OR(95%CI)	p
Sex	Boy	27(16.4)	1	0.350	26(15.8)	1	0.881
	Girl	21(12.7)	0.75(0.40–1.38)		27(16.4)	1.05(0.58–1.88)	
Grade	3rd	6(6.2)	1	0.010^**^	14(14.4)	1	0.491
	4th	11(13.3)	2.32(0.82–6.57)		11(13.3)	0.91(0.39–2.12)	
	5th	31(20.7)	3.95(1.58–9.87)		28(18.7)	1.36(0.68–2.74)	
Age	8–9 y	11(10.6)	1	0.162	15(14.4)	1	0.561
	10–11 y	36(16.5)	1.67(0.81–3.44)		37(17)	1.21(0.63–2.33)	
Being trapped	No	28(12.6)	1	0.156	37(16.7)	1	0.667
	Yes	20(18.5)	1.58(0.84–2.95)		16(14.8)	0.87(0.46–1.65)	
Being injured	No	16(8.3)	1	0.000^***^	24(12.4)	1	0.035^*^
	Yes	32(23.4)	3.37(1.77–6.44)		29(21.2)	1.89(1.05–3.42)	
Bereavement	No	2(2.8)	1	0.006^**^	6(8.3)	1	0.049^*^
	Yes	46(17.8)	7.59(1.79–32.09)		47(18.2)	2.45(1.00–5.99)	
Witness death	No	17(8.9)	1	0.001^***^	26(13.6)	1	0.149
	Yes	31(22.5)	2.97(1.56–5.62)		27(19.6)	1.54(0.86–2.78)	
Extreme fear	No	10(7.4)	1	0.003^**^	6(4.4)	1	<0.001
	Yes	38(19.6)	3.07(1.47–6.40)		47(24.2)	6.93(2.87–16.73)	

Abbreviation: No. = number.

Depression 1 and Depression 2 represent depression at 6-month and depression at 12-month, respectively.

**Table 5:** Bivariate analysis of effects of demographic and trauma exposure variables on symptoms of PTSD at 6-month and 12-month after the Sichuan earthquake.

	PTSD 1				PTSD 2		
	Variable	No.(%)	OR(95%CI)	p	No.(%)	OR(95%CI)	p
Sex	Boy	18(10.9)	1	0.862	25(15.2)	1	0.333
	Girl	19(11.5)	1.06(0.53–2.11)		19(11.5)	0.73(0.38–1.38)	
Grade	3rd	4(4.1)	1	0.047^*^	12(12.4)	1	0.808
	4th	12(14.5)	3.93(1.22–12.70)		10(12)	0.97(0.39–2.37)	
	5th	21(14)	3.79(1.25–11.39)		22(14.7)	1.22(0.57–2.59)	
Age	8–9 y	12(11.5)	1	0.985	12(11.5)	1	0.444
	10–11 y	25(11.5)	0.99(0.48–2.06)		32(14.7)	1.32(0.65–2.68)	
Being trapped	No	23(10.4)	1	0.483	27(12.2)	1	0.371
	Yes	14(13)	1.29(0.64–2.62)		17(15.7)	1.35(0.70–2.60)	
Being injured	No	17(8.8)	1	0.104	21(10.9)	1	0.122
	Yes	20(14.6)	1.77(0.89–3.52)		23(16.8)	1.65(0.87–3.12)	
Bereavement	No	1(1.4)	1	0.017^*^	5(6.9)	1	0.072
	Yes	36(14)	11.51(1.55–85.49)		39(15.1)	2.39(0.90–6.30)	
Witness death	No	13(6.8)	1	0.004^**^	21(11)	1	0.138
	Yes	24(17.4)	2.88(1.41–5.89)		23(16.7)	1.62(0.86–3.06)	
Extreme fear	No	10(7.4)	1	0.064	11(8.1)	1	0.022^*^
	Yes	27(13.9)	2.04(0.95–4.36)		33(17)	2.33(1.13–4.79)	

Abbreviation: No. = number.

PTSD 1 and PTSD 2 represent PTSD at 6-month and PTSD at 12-month, respectively.

In the first survey (6 months after the earthquake), symptoms of anxiety, depression and PTSD were significantly more common among students of grades 4 and 5 than those in grade 3. Children who experienced physical injuries and witnessed death had higher prevalence rates of anxiety symptoms, while those who had physical injuries, bereavement, witnessing death and extreme fear had higher prevalence rates on the symptoms of depression and PTSD.

At the time of follow-up survey, children who witnessed death and experienced extreme fear had higher prevalent rates on the symptoms of anxiety; those who had physical injuries, bereavement and extreme fear had higher prevalent rates on the symptoms of depression; and those who had extreme fear had higher prevalent rates on the symptoms of PTSD.

In the multivariate analysis for the first survey, the variable of grade was still significantly associated with the prevalence rates of traumatic symptoms. Students of grades 4 and 5 had higher prevalence rates than those in grade 3 on the symptoms of anxiety (odds ratio [OR](95%CI): 4^th^:2.08 (0.94, 4.58), 5^th^: 2.55(1.28, 5.11), *p* = 0.029), depression (OR(95%CI): 4^th^:2.55(0.86, 7.57), 5^th^:3.49(1.35, 9.02), *p* = 0.035) and PTSD (OR(95%CI): 4^th^:4.15(1.21, 13.26), 5^th^:3.57(1.13, 10.64), *p* = 0.045). Children who had physical injuries (OR(95%CI): 1.87(1.06, 3.30), *p* = 0.032) had higher prevalence rates of anxiety symptoms; those who experienced bereavement (OR(95%CI): 5.14(1.18, 22.38), *p* = 0.029)and extreme fear (OR(95%CI): 2.31(1.07, 5.01), *p* = 0.033)had higher prevalence rates on the symptoms of depression; those who experienced bereavement (OR(95%CI): 9.30(1.24, 70.03), *p* = 0.003)and witnessed death (OR(95%CI): 2.43(1.17, 5.06), *p* = 0.018)had higher prevalence rates on the symptoms of PTSD.

The results of multivariate analysis for the second survey showed that, among trauma exposure variables, only extreme fear has significant effects on symptoms of anxiety (OR: 4.20, 95% CI, 2.19–8.06; *p* = 0.000), depression (OR: 6.39, 95% CI, 2.62–15.58; *p* = 0.000) and PTSD (OR: 2.33, 95% CI, 1.13–4.79; *p* = 0.022). Demographic variables have not significant effects on the prevalent rates of the mental health problems.

## Discussion

The current study is concerned with the changes of prevalence of probable anxiety, depression and PTSD among children 6 months and 12 months after the Sichuan earthquake in China and the effects of the earthquake on children's mental health problems.

Based on the assessments, the prevalence rates of the mental health problems stayed relatively similar. The percentage of probable positive anxiety was slightly higher at the point of 6-month than that at 12-month while the percentages of probable positive depression and PTSD were higher at 12-month than those at 6-month, but all these differences were not statistically significant. These results were consistent with those from other studies. For example, no significant differences were found on the prevalence rates of depression and PTSD symptoms between 8 weeks and 9 months after tsunami [9]. The severity of symptoms also got the same appearance with the prevalence rates. As Table 2 showed, the results for symptoms of depression and PTSD were statistically significant, but the difference is small enough to be utterly unimportant. The result that the severity of symptoms of anxiety, depression and PTSD at the time point of 12 months was similar with those in 6 months after the earthquake described the changes in more detail.

Continuous high rates of mental health problems and steady severity imply that these problems have a chronic course [1], [10], [15]. These results indicate that, during those days of the second survey, grief and desperation induced by the tragic earthquake still overwhelmingly affected those children. In addition, the fact that ongoing housing and other types of stress associated with reduced resources following the earthquake may also account for children's distress. These phenomena show that post-earthquake mental health problems in children may remain high for a long period. Recovering from natural disasters is often a drawn-out process [11], [16].

Among the demographic variables of interest, the result was congruent with a study in which no significant age difference existed [9], while it was found that 6 months after the earthquake, symptoms of anxiety, depression and PTSD were significantly more common among students in grades 4 and 5 than those in grade 3, which was seemingly incongruent with the result about age. Other studies showed that younger children were more susceptible to the negative consequences of multiple risks than older ones [4], [5], Traumas in these studies were often caused by other people, such as family violence or sexual victimization rather than earthquake or hurricane. The current study adopted the variable of grade which is highly correlated with age. This may be related to the development of intellectual or cognition abilities [17], [18]. To our knowledge, the study reported here is the first to provide the evidence that schooling effect is different from any age effect. The possible reason of the significant schooling effect appeared at 6 months after the earthquake could be that children in grade 3 were too young to fully realize the effects of the tragedy. In addition, we do not know the difference in experience of the different grades either at the time of the earthquake or subsequently, more surveys should be conducted to observe and clarify the schooling effect.

Few follow-up studies have documented the complex relationships between exposure factors and psychological sequences after natural disasters in children. The results about exposure factors of this study were congruent with those found in previous studies in adults, which reported that individuals who experienced more traumatic stresses were more likely to develop mental health problems, such as PTSD, depression and anxiety [19], [20]. We found that personal injury and witnessing death were independent risk factors for anxiety, while personal injury, bereavement, witnessing death and extreme fear were independent risk factors for symptoms of depression and PTSD. Compared with children without experiencing bereavement, those who had the experiences of bereavement had 11 times higher risk for PTSD and 8 times for depression. Similarly, the experience of exposure to death has been considered an important factor in the development of psychological morbidity [9], [21]. With regard to the changes of effective exposure variables, findings in the survey showed that extreme fear played an important role on the symptoms of anxiety, depression and PTSD one year after the earthquake. As is well-known, a typical mental health intervention begins with an assessment of what happened during the crisis and the individual's responses to it, so these results may be useful for psychologists and social workers to screen and identify high-risk individuals with those exposure factors and then provide appropriate mental health interventions to reduce the intensity of their mental reactions to the earthquake.

Several limitations to this study should be noted. First, the generalizability of our findings to all children survivors of the Sichuan earthquake is limited by selecting a camp primary school in Beichuan County where the damages were heavier than other places. We did not sample a non-camp control group for a comparison. Second, data were collected through self-report questionnaires, which provide limited room for clinical clarification. The retrospective component of the study may inevitably have caused some potential bias and confounding factors. Third, some trauma exposure variables which may be important contributors to outcomes were not sufficiently detailed, such as the nature of the bereavement (who was lost), the severity of the injuries (who required surgery or extensive hospitalizations). Fourth, there may be several additional trauma exposure indictors that were not included in this study.

Even with these limitations, this study may have made several contributions. First, this study expands our knowledge on posttraumatic symptoms and related risk factors in children following a very destructive natural disaster in non-Western communities. Second, we found that the prevalence rates of depression and PTSD stayed relatively similar in 6 months and 12 months after a very destructive earthquake. Third, significant schooling effect indicated that learning experience in school could provide more information than age. Finally, in children, disaster-related experiences, especially exposure to death and initial extreme fear are important on initiating and maintaining mental health problems. We should pay more attention to this in future research.
